# Unraveling Leukocyte Profile Shifts and Platelet Dynamics Following Leukoreduced Packed Red Cell Transfusions in Very Low Birth Weight Preterm Neonates

**DOI:** 10.7759/cureus.44900

**Published:** 2023-09-08

**Authors:** Palanikumar Balasundaram, Sharef Waadallah Al-Mulaabed, Kim Roger

**Affiliations:** 1 Division of Neonatology, Department of Pediatrics, Javon Bea Hospital, Mercy Health, Rockford, USA; 2 Center for Digestive Health and Nutrition, Arnold Palmer Hospital for Children, Orlando, USA; 3 Division of Neonatology, Brookdale University Hospital Medical Center, Brooklyn, USA

**Keywords:** vlbw, preterm, neonatal intensive care unit (nicu), leukoreduced, platelet, leukocyte count, packed red blood cells ( prbc)

## Abstract

Background

Packed red blood cell (PRBC) transfusions are routine in neonatal care and the most common blood product administered to sick neonates. However, their impact on leukocyte and platelet profiles in very low birth weight (VLBW) preterm infants remains largely unexplored. This study examines leukocyte profile shifts and platelet dynamics following leukoreduced PRBC transfusions in VLBW preterm infants, offering insights to improve neonatal care and reduce unnecessary interventions.

Methods

The study utilized a retrospective cohort design within a single center, focusing on VLBW preterm infants who received PRBC transfusions at a level 3 NICU between January 2014 and June 2019. Data collection encompassed white blood cell (WBC) and platelet count measurements taken 24 hours before and up to 72 hours after PRBC transfusion. Neonates lacking complete blood count (CBC) data within the 72-hour post-transfusion window were excluded. A subgroup analysis distinguished the outcome between the initial PRBC transfusion and subsequent ones. The statistical significance of pre- and post-transfusion laboratory data was determined using the Wilcoxon signed ranks test and paired T-test.

Results

A cohort of 108 VLBW preterm infants who underwent a total of 402 PRBC transfusions was included in the analysis. The subjects exhibited a mean gestational age of 27.2 ± 2.5 weeks and a mean birth weight of 913 ± 264 grams. Analysis of pre- and post-transfusion data revealed no significant differences in total white blood cell count (WBC), absolute neutrophil count (ANC), absolute monocyte count (AMC), absolute eosinophil count, and absolute lymphocyte count. Notably, the platelet count was significantly decreased in the post-transfusion group (p < 0.001). In a subset analysis limited to the first-time transfusions among the 108 infants, a statistically significant increase was observed in total WBC, AMC, and ANC following transfusion.

Conclusions

The findings of this study highlight that PRBC transfusions can prompt an increase in neutrophils, monocytes, and eosinophils, coupled with a decline in platelet counts, all within a 72-hour window post-transfusion. Notably, these changes were predominantly discernible after the initial transfusion, with subsequent transfusions demonstrating consistency, except for the observed platelet count reduction. Recognizing these patterns could prove instrumental in averting undue investigations for suspected sepsis, particularly following the initial transfusion event. However, further in-depth investigations are necessary to uncover the underlying factors responsible for the shifts in leukocyte and platelet profiles triggered by PRBC transfusions.

## Introduction

Blood transfusions are a common intervention in premature infants, with more than 80% of very low birth weight (VLBW) infants receiving at least one transfusion during their hospital stay [1,2]. Packed red blood cells (PRBC) are the most frequently administered blood product to sick neonates. The presence of leukocytes within PRBCs is responsible for adverse reactions linked to blood transfusion. However, many countries now routinely employ leukoreduction before red cell storage. Leukoreduction seeks to reduce transfusion-related reactions by removing donor leukocytes from PRBC units through filtration while ensuring the residual leukocyte count remains below 5.0 x 10^6^ per each whole blood, compliant with current Food and Drug Administration (FDA) regulations [3]. Leukocytes, also known as white blood cells (WBCs), play a pivotal role in the body's defense against invading pathogens [4]. The origin of leukocytes commences in the liver at approximately five weeks of gestation [5]. Apart from WBC counts, the absolute neutrophil count (ANC), immature-to-total neutrophil ratio (I/T ratio), and platelet counts are also extensively employed as possible indicators of infection [6].

Insights from studies involving adults propose a connection between increased morbidity and mortality in critically ill adults after blood transfusion, which raises the possibility of heightened pro-inflammatory immunomodulation [7]. This speculation prompts the exploration of whether changes in circulating WBCs may follow PRBC transfusions. Several works of literature have shown that PRBC transfusions were associated with transient leucocytosis in adult recipients [8-10]. Nonetheless, significant research gaps exist, including the scarcity of large-scale studies addressing whether transfusions of leukoreduced red cells lead to leukocytosis in anemic, critically ill patients in intensive care. Furthermore, studies focusing on leukocyte and platelet profile shifts in premature neonates after leukoreduced PRBC are limited in number [11].

The awareness of post-transfusion leukocytosis can prevent unnecessary investigations and therapies fueled by misguided suspicions of sepsis. This study aims to analyze the changes in leukocyte and platelet profile following a transfusion of leukoreduced red blood cells in VLBW and preterm neonates.

## Materials and methods

Study cohort

This retrospective analysis focused on a cohort of VLBW and preterm neonates who received PRBC transfusions at an urban level 3 NICU between January 2014 and May 2019. Neonates with a complete blood count (CBC) within the 72-hour post-transfusion window following the PRBC transfusion were included in the analysis. The study's exclusion criteria covered several factors, such as neonates transferred to another facility post-transfusion, neonates who unfortunately passed away after the transfusion, cases with incomplete medical chart data, and neonates who received additional transfusions between the two CBC sample collections.

General clinical practice

Each PRBC transfusion in this study involved cytomegalovirus-negative, irradiated, and sickle cell-negative PRBCs. Prior to storage, these PRBC units had undergone leukoreduction. Each neonate received PRBCs at a volume ranging from 10 to 20 ml per kg, with an infusion duration spanning two to four hours. Infants undergoing the transfusion procedure were placed on a nil per oral status. Before 2020, we routinely checked hematocrit post-transfusion for all neonates within 72 hours. However, this practice has since been discontinued, and we used the collected CBC data during that period to analyze our research outcomes and gather valuable insights.

Data collection

The study utilized electronic medical records to gather pertinent neonatal demographic characteristics and laboratory data. Demographic data encompassed gestational age (GA), birth weight (BW), and sex. The laboratory data comprised WBC counts, ANC, absolute eosinophil count (AEC), absolute monocyte count (AMC), absolute lymphocyte count (ALC), and platelet counts. These laboratory values were measured 24 hours before the transfusion and 24 to 72 hours after. All study-related data were collected, compiled, and securely stored in a password-protected folder within a secure network environment to maintain confidentiality.

Definitions

GA was determined according to the obstetrician's standard of care. Neonates born at less than 37 weeks of gestation were classified as preterm neonates. Infants with a birth weight of less than 1500 grams were categorized as VLBW neonates.

Statistical analysis

Data were analyzed using the Statistical Package for the Social Sciences (SPSS) software, version 20 (IBM Corp., Armonk, NY). Descriptive statistics, including mean (± standard deviation) or median (interquartile range), were employed based on the distribution of data. Statistical significance of laboratory data before and after transfusion was determined using appropriate tests such as the Wilcoxon signed ranks test and paired T-test. A two-sided p-value of less than 0.05 was considered statistically significant. A subgroup analysis was conducted to differentiate outcomes between the initial PRBC transfusion and subsequent transfusions.

Ethical considerations

The study protocol underwent review and approval by our institution's institutional review board. Considering the study's retrospective design, the requirement for consent was waived.

## Results

Demographic characteristics

A total of 108 VLBW and preterm infants were included in the study. The demographic characteristics of the subjects are summarized in Table 1. The infants exhibited a mean gestational age of 27.2 weeks, and their average birth weight was 913 grams. Among them, 58 infants (54%) were male.

**Table 1:** Demographic characteristics of the study population (n=108) n - number, SD - standard deviation

Demographic characteristics	Values
Gestational age in weeks, Mean (±SD)	27.2 (±2.5)
Birth weight in grams, Mean (±SD)	913 (± 264)
Sex	
Male, n (%)	58 (54%)
Female, n (%)	50 (46%)

Laboratory parameters before and after blood transfusions

Table 2 provides a detailed overview of the changes in laboratory parameters before and after blood transfusions across the entire cohort of 108 studied subjects, encompassing a total of 402 transfusion encounters. The analysis revealed no statistically significant differences in the median (interquartile range) values of various parameters before and after transfusion. Specifically, the WBC (×10^9^/L) showed consistent values before and after transfusion, with medians of 17.1 (11.7-23.3) and 17.1 (12.6-22.9), respectively (p = 0.625). Similarly, no significant differences were observed in the ANC, absolute monocyte count, absolute lymphocyte count, and absolute eosinophil count. However, the platelet count (×10^9^/L) demonstrated a statistically significant decrease post-transfusion, with median values dropping from 205 (128-300) to 181 (118-272) (p < 0.001).

**Table 2:** Change of laboratory parameters before and after blood transfusions This table displays changes in laboratory parameters before and after transfusions in 108 subjects (402 transfusion encounters). IQR - interquartile range

Laboratory finding	Before transfusion	After transfusion	P value
WBC (×10^9^/L), Median (IQR)	17.1 (11.7-23.3)	17.1 (12.6-22.9)	0.625
Absolute neutrophil count (×10^9^/L), Median (IQR)	7.5 (4.1-11.9)	7.2 (4.5-11.8)	0.509
Absolute monocyte count (×10^9^/L), Median (IQR)	1.9 (1.2-3.0)	2.0 (1.2-3.1)	0.367
Absolute lymphocyte count (×10^9^/L), Median (IQR)	4.5 (3.2-6.2)	4.6 (2.9-6.1)	0.739
Absolute eosinophils count (×10^9^/L), Median (IQR)	0.6 (0.2-1.1)	0.5 (0.3-1.2)	0.724
Platelet count (× 10^9^/L), Median (IQR)	205 (128-300)	181 (118-272)	< 0.001

Laboratory parameters before and after first blood transfusions

Table 3 provides a focused view of the changes in laboratory parameters, specifically before and after the first-time blood transfusions within the cohort of 108 infants, encompassing a total of 108 transfusion encounters. Among these infants, the analysis revealed significant changes in certain parameters. The WBC (×109/L) exhibited a significant increase post-transfusion, rising from a median of 12.7 (7.7-21.2) before to 16.0 (9.0-24.7) after the transfusion (p = 0.002). This trend was echoed in the ANC (×109/L), which demonstrated a significant increase from 4.7 (2.8-10.6) to 7.7 (3.2-13.3) (p = 0.004). Additionally, the absolute monocyte count showed a significant increase post-transfusion, with median values increasing from 1.4 (0.8-2.2) to 1.6 (1.0-2.5) (p = 0.004). There were no statistically significant differences in the mean absolute lymphocyte count, absolute eosinophil count, or platelet count.

**Table 3:** Change of laboratory parameters before and after first-time blood transfusions This table illustrates changes in laboratory parameters before and after first-time blood transfusions among 108 subjects (108 transfusion encounters). SD - standard deviation, IQR - interquartile range

Laboratory finding	Before transfusion	After transfusion	P value
WBC (×10^9^/L), Median (IQR)	12.7 (7.7-21.2)	16.0 (9.0-24.7)	0.002
Absolute neutrophil count (×10^9^/L), Median (IQR)	4.7 (2.8-10.6)	7.7 (3.2-13.3)	0.004
Absolute monocyte count (×10^9^/L), Median (IQR)	1.4 (0.8-2.2)	1.6 (1.0-2.5)	0.004
Absolute lymphocyte count (×10^9^/L), Mean (±SD)	4.5 (±2.3)	4.4 (±2.1	0.825
Absolute eosinophils count (×10^9^/L), Median (IQR)	0.3 (0.1-0.6)	0.3 (0.2-0.8)	0.355
Platelet count (× 10^9^/L), Mean (±SD)	235 (±118)	226 (±133)	0.007

**Figure 1:**
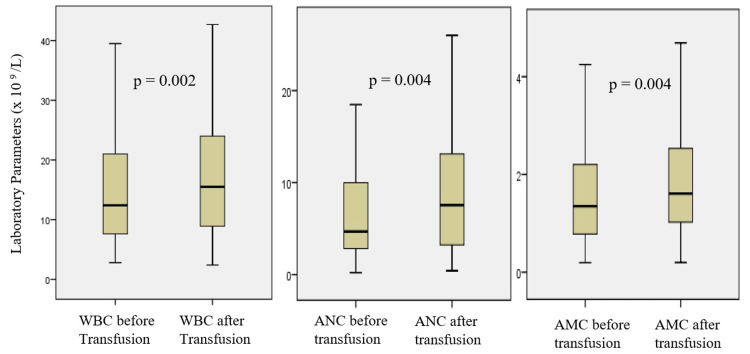
Change of laboratory parameters before and after first-time blood transfusions WBC – white blood cell count, AMC – absolute monocyte count, ANC – absolute neutrophil count

Figure 1 presents a comprehensive visualization of the alterations in laboratory parameters before and after the initial PRBC transfusions. The data are depicted using box and whisker plots, which provide valuable insights into the distribution and central tendency of the measurements. The parameters of interest include WBC, AMC, and ANC. The boxes in the plots represent the data's interquartile range (IQR), with the median indicated by a line across the box. The key feature highlighted by this figure is the statistically significant increase in WBC, ANC, and AMC after the first blood transfusions.

## Discussion

Blood transfusions play a critical role in the care of premature infants, particularly those with VLBW [12]. Despite their widespread use, there is a growing concern regarding the potential immunomodulatory effects of transfusions, particularly with PRBCs [13,14]. Until now, only a few studies have examined the correlation between PRBC transfusion and WBC indices in preterm and VLBW infants [15,16].

The observed elevation in total WBC counts, neutrophils, and monocytes within 72 hours after transfusion in this study suggests a complex interplay between transfusion and immune response. Notably, the elevation was more pronounced after the initial transfusion, implying a primed immune response that becomes more regulated in subsequent transfusions. This observation aligns with adult study findings suggesting a link between transfusions and transient leukocytosis. The observed increase in WBC count following transfusion in our study could be attributed to post-transfusion immunomodulation. Previous research has indicated a significant rise in pro-inflammatory cytokines such as Interleukin (IL)-1β, IL-6, IL-8, tumor necrosis factor-α, interferon-γ, IL-17, and intracellular adhesion molecule-1 levels after exposure to packed RBCs [17-19]. Certain studies suggest that pre-storage leukoreduction can notably reduce the buildup of pro-inflammatory cytokines [13,20,21]. However, leukoreduced PRBCs may still contain some pro-inflammatory mediators that could contribute to the post-transfusion changes in WBCs [14]. It's likely that the influence of leukoreduced PRBCs on WBC profiles is transient, which might explain why this effect is not consistently observed in subsequent transfusions.

The observed reduction in platelet count following transfusion in our study could likely be attributed to the phenomenon of dilution and an associated increase in platelet aggregation, as indicated by in vitro transfusion studies [22,23]. When blood is transfused, the introduction of additional fluid volume from the transfused blood can lead to a dilution effect, which might cause a decrease in the concentration of platelets per unit volume of blood. This dilution effect can consequently result in a lower platelet count in the recipient's bloodstream. Furthermore, the process of platelet aggregation, where platelets clump together, can be triggered as a response to the changes in the blood environment during and after transfusion. This aggregation can temporarily reduce the actual count of individual platelets while maintaining their functionality as a collective entity. Dilutional thrombocytopenia has been documented in adults during massive transfusions [24] and neonates during exchange transfusions [25]. It is plausible that a similar mechanism may contribute to the observed decline in platelet count following PRBC transfusion in preterm neonates.

However, it's worth noting that this reduction in platelet count was not observed after the initial transfusion. This discrepancy might arise due to various factors. First, the initial transfusion might have a different impact on platelet count than subsequent transfusions due to factors such as the recipient's physiological response, dilution, and degree of platelet aggregation [24,25]. Second, the initial transfusion might have triggered other compensatory mechanisms that counteracted the platelet count reduction. Further research is needed to elucidate the exact mechanisms underlying these observations and better understand the dynamics of platelet count changes following transfusion events.

Despite the valuable insights gained from this study regarding the impact of PRBC transfusions on leukocyte and platelet profiles in premature and VLBW neonates, several limitations should be acknowledged. The study's retrospective nature introduces potential biases stemming from reliance on electronic medical records, potentially leading to inconsistencies or missing data. Furthermore, the study's relatively small sample size and single-center nature could limit the generalizability of the findings across diverse neonatal populations. The absence of a control group that did not receive PRBC transfusions hinders establishing a direct causal relationship between transfusions and observed profile changes. The study's confined 72-hour observation window might not capture potential longer-term effects or delayed responses. The omission of other relevant variables, such as cytokine levels and coagulation parameters, also limits the depth of understanding. Due to the limitation of an insufficient sample size, we were unable to stratify the results based on the timing of post-transfusion CBC collection. In light of these limitations, future research with larger sample sizes, prospective designs, and more comprehensive assessments is essential for a more nuanced comprehension of the intricate interactions between PRBC transfusions and neonatal immune responses.

## Conclusions

The findings of this study underscore the potential impact of PRBC transfusions on the dynamics of key blood cell populations. Specifically, our findings reveal an elevation in WBC, neutrophils, and monocytes and a reduction in platelet counts within 72 hours following transfusion. Remarkably, these alterations were most prominent following the initial transfusion, suggesting a distinctive response that tends to stabilize in subsequent transfusions, except for the observed platelet count reduction. Recognizing these post-transfusion patterns is crucial for appropriate clinical interpretation and decision-making, preventing unnecessary interventions arising from misinterpreted physiological responses. While we may not understand all mechanisms for the increase in WBC count or decreased platelet count post-transfusion, we do know the phenomenon exists, and we should avoid labeling all neonates with leukocytosis and thrombocytopenia in the NICU as infected. However, further in-depth investigations are necessary to uncover the underlying factors responsible for the shifts in leukocyte and platelet profiles triggered by PRBC transfusions. 
